# Supplementary material for: Doxycycline Inhibition of a Pseudotyped Virus Transduction Does Not Translate to Inhibition of SARS-CoV-2 Infectivity
Source: Viruses. 2021 Sep 1;13(9):1745. doi: 10.3390/v13091745 (PMC8473150; doi:10.3390/v13091745)
Supplement: Supplementary file 1 [file viruses-13-01745-s001.zip › viruses-1351739-supplementary.pdf]

Supplementary Material

S1 Appendix

Image analysis

Setting a fixed threshold for all the images, the number of GFP-positive cells was calculated with the function Analyze particles, using the watershed setting to segment cell clusters in the most accurate manner. The median function and Analyze particles set-up were used to exclude noise and non-specific debris from the count. The macro used to analyze the images acquired with ZOE™ Fluorescent Cell Imager is reported below:

```
run(“8-bit”);
setAutoThreshold(“Default dark”);
//run(“Threshold…”);
setThreshold(11,255);
setOption(“BlackBackground”, true);
run(“Convert to Mask”);
run(“Median…”, “radius=3”);
run(“Watershed”);
run(“Analyze Particles…”, “size=500-Infinity show=Outlines display summarize”);
```

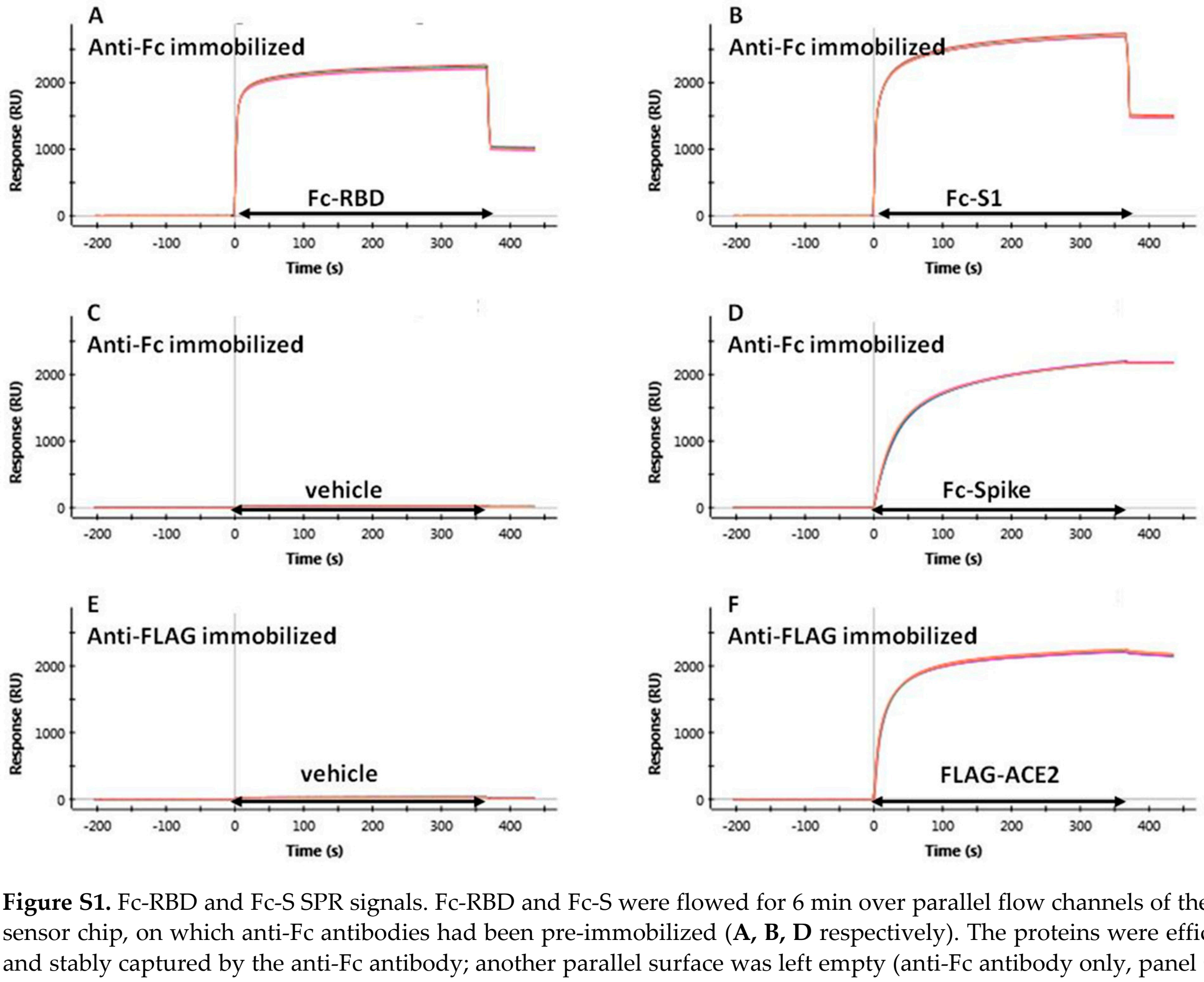

Figure S1. Fc-RBD and Fc-S SPR signals. Fc-RBD and Fc-S were flowed for 6 min over parallel flow channels of the same sensor chip, on which anti-Fc antibodies had been pre-immobilized (**A, B, D** respectively). The proteins were efficiently and stably captured by the anti-Fc antibody; another parallel surface was left empty (anti-Fc antibody only, panel **C**), for use as reference. Flag-ACE2 was flowed for 6 min over another parallel flow channel of the same sensor chip, on which an anti-Flag antibody had been pre-immobilized (panel **F**). ACE2 was efficiently and stably captured by the anti-Flag antibody; another parallel surface was left empty (anti-Flagantibody only, panel **E**), for use as reference.

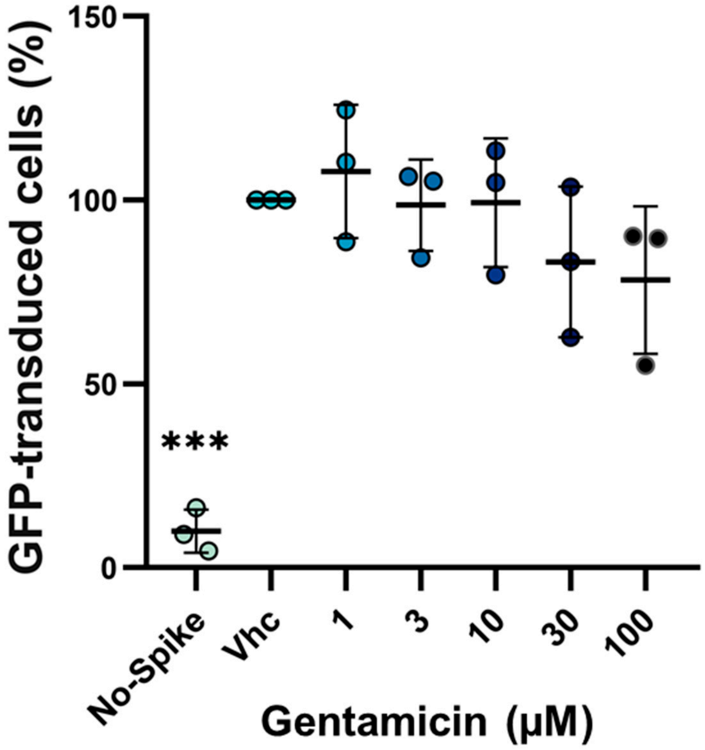

Figure S2. Dose-response effect of gentamicin in VeroE6 cells. The y-axis showed the mean± SD percentage of GFP-transduced cells in relation to control cells treated with vehicle alone (Vhc). The percentage of Vero E6 cells transduced with the retroviral vector without SARS-CoV-2 S protein (No-Spike) is reported as negative control.

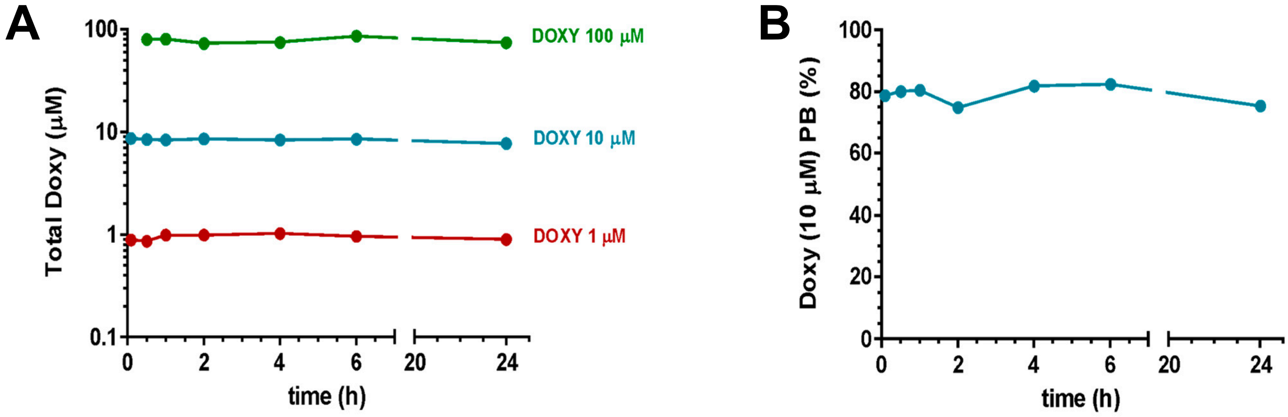

Figure S3. Binding of doxycycline to BSA. Ten and 100 μM in DMEM medium added to HEK293-ACE2 cells seeded on 96-well plates (as in the transduction assay). Total **(A)** and BSA-bound doxycycline (**B,** for 10 μM only) were measured after 5 min and 0.5 1, 2, 4, 6 and 24h of incubation. Each point represents the mean of two independent measures.

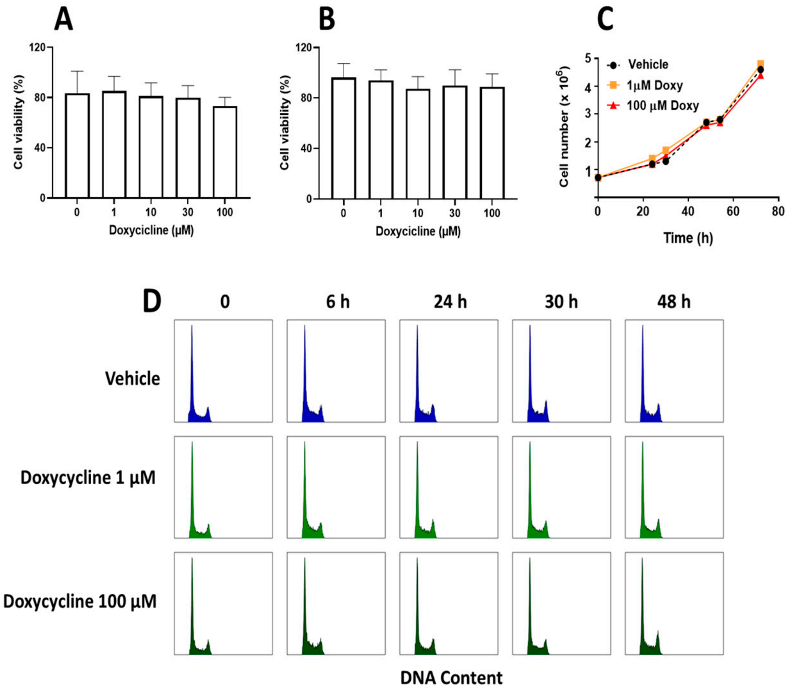

Figure S4. Doxycycline did not affect cell viability and proliferation. Cell viability of (**A**) Vero E6 and (**B**) HEK293-ACE2 cells treated with different concentrations of doxycycline, in the MTT assay. Data are the mean ± SD percentage of viable cells, in relation to control cells (treated with vehicle only, no doxycycline), from three independent experiments. (**C**) Cell proliferation assay for HEK293-ACE2 cells, days 1–3, investigating the effects of 1 and 100 µM doxycycline (Doxy). (**D**) Cell cycle progression assay of HEK293-ACE2 cells treated with 1 or 100 µM doxycycline for 6 h up to 48 h.

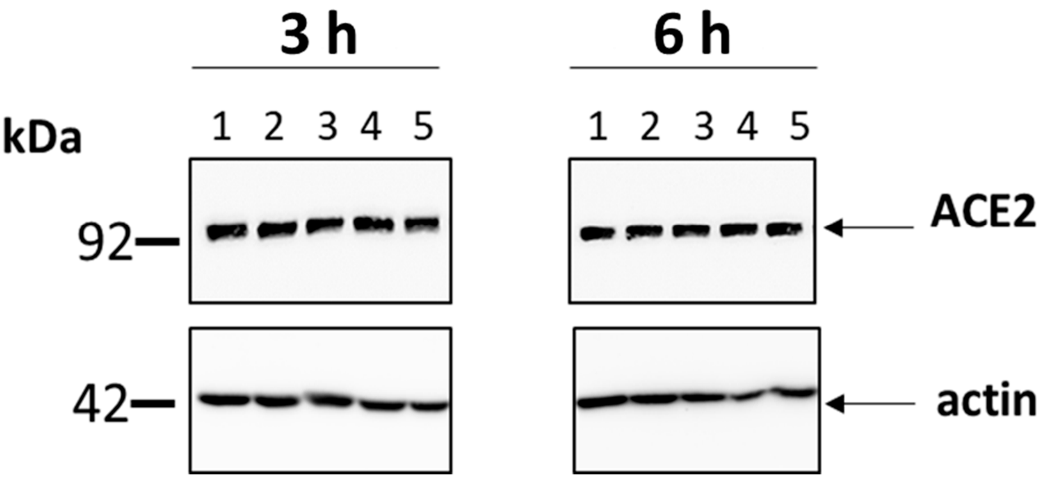

Figure S5. Effect of doxycycline on ACE2 expression. HEK293-ACE2 cells were treated for 3 h or 6 h with 0.1 µM (lane 2) 1.0 µM (lane 3) or 100 µM (lane 4) doxycycline. Control cells were treated with the same volume of Milli-Q water (lanes 1 and 5). Cells were then lysed and analyzed by western blotting. Signals were detected using a specific anti-ACE2 primary antibody and relevant HRP-coupled secondary antibodies and revealed using a ChemiDoc Touch Imaging System. Western blot images are representative examples of three separate experiments.

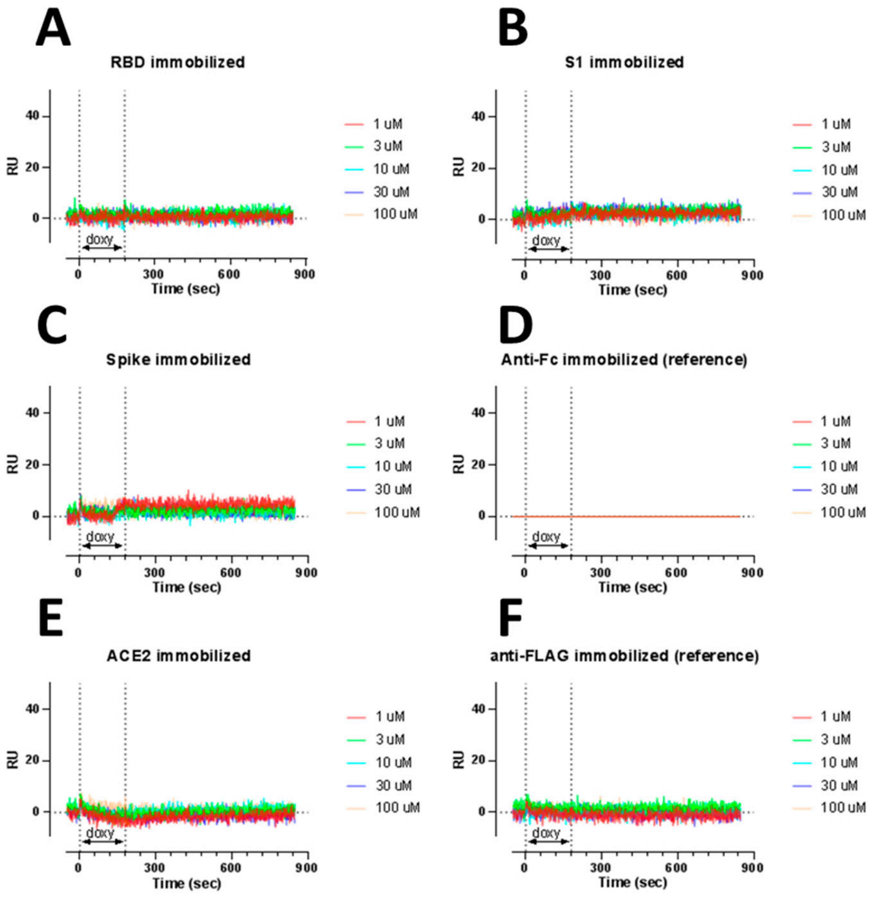

Figure S6. Surface plasmon resonance shows no direct binding of doxycycline to (A) RBD, (B) S1, (C) Spike protein and (E) ACE2 proteins. Doxycycline was flowed for 180 secs (as indicated) over parallel flow channels of the same sensor chip on which we had previously captured Fc-RBD, Fc-S1 and Fc-S (by anti-Fc antibody) or Flag-ACE2. Two flow channels were only coated with (**D**) anti-Fc or (**F**) anti-Flag antibodies. Doxycycline was injected at concentrations from 1 to 100 μM, in PBST pH 7.4 with short dissociation times between, without regeneration steps. The graphs show the sensorgrams obtained after subtraction of the SPR signal on the anti-Fc antibody, used as reference (**C**). The sensorgrams shows there was no "specific" binding to the protein of interest, even at the highest concentration (100 μM).
